# Cardiac examination and the effect of dual-processing instruction in a cardiopulmonary simulator

**DOI:** 10.1007/s10459-012-9388-6

**Published:** 2012-06-21

**Authors:** Matt Sibbald, James McKinney, Rodrigo B. Cavalcanti, Eric Yu, David A. Wood, Parvathy Nair, Kevin W. Eva, Rose Hatala

**Affiliations:** 1 Dr. Ho Ping Kong Center for Excellence in Education and Practice, University Health Network-Western Division, 399 Bathurst Street, 8 East 427B, Toronto, ON M5T 2S8 Canada; 2 Department of Medicine, University of British Columbia, Vancouver, BC Canada; 3 Division of General Internal Medicine, University of Toronto, Toronto, ON Canada; 4 Division of Cardiology, University of Toronto, Toronto, ON Canada; 5 Division of Cardiology, University of British Columbia, Vancouver, BC Canada; 6 Division of General Internal Medicine, University of British Columbia, Vancouver, BC Canada

**Keywords:** Dual-processing, Cardiac physical exam, Diagnostic error, Clinical instruction, Systems processing

## Abstract

Use of dual-processing has been widely touted as a strategy to reduce diagnostic error in clinical medicine. However, this strategy has not been tested among medical trainees with complex diagnostic problems. We sought to determine whether dual-processing instruction could reduce diagnostic error across a spectrum of experience with trainees undertaking cardiac physical exam. Three experiments were conducted using a similar design to teach cardiac physical exam using a cardiopulmonary simulator. One experiment was conducted in each of three groups: experienced, intermediate and novice trainees. In all three experiments, participants were randomized to receive undirected or dual-processing verbal instruction during teaching, practice and testing phases. When tested, dual-processing instruction did not change the probability assigned to the correct diagnosis in any of the three experiments. Among intermediates, there was an apparent interaction between the diagnosis tested and the effect of dual-processing instruction. Among relative novices, dual processing instruction may have dampened the harmful effect of a bias away from the correct diagnosis. Further work is needed to define the role of dual-processing instruction to reduce cognitive error. This study suggests that it cannot be blindly applied to complex diagnostic problems such as cardiac physical exam.

## Background

Diagnostic error is common within clinical medicine (Graber 2005). While difficult to ascribe to isolated causes, many of these errors involve mistakes of cognitive processing (Graber et al. 2005). In a recent systematic review, 65 % of errors by internal medicine physicians were related to cognitive factors (Graber et al. 2005). As a result, many have suggested teaching metacognition to avoid cognitive errors. However, there is little empiric data to support this intervention (Norman 2005).

Understanding cognitive errors requires a broader understanding of clinician thinking. From a cognitive psychology perspective, clinical thinking within medicine can be divided into two distinct reasoning processes: system 1 or non-analytic reasoning (e.g., pattern recognition) and system 2 or analytic reasoning (e.g., careful and deliberate weighing of features and diagnoses) (Kahneman 2011; Norman and Eva 2010; Eva 2005). The predominant strategy used by an individual clinician is often linked to clinical experience. More experienced practitioners appear to rely to a greater extent on non-analytic reasoning than novices (Eva and Cunnington 2006). This is reflected in the differing sources of diagnostic errors between novices and practicing physicians. Using written clinical problems, Groves et al. (2003) found that errors made by experienced physicians were mostly attributable to misidentification of critical features in contrast to intermediates who more often failed to generate the correct hypothesis (Groves et al. 2003).

More recently, interest has emerged in cognitive flexibility: the ability of the clinician to move between multiple reasoning strategies (Eva 2005). The central hypothesis is that combining fast, automatic, unconscious processes (system 1) with slower, effortful, conscious processes (system 2) will reduce diagnostic error relative to relying on either set of processes in isolation (Evans 2008).

Within clinical medicine, the merit of an intervention instructing diagnosticians to use a dual-processing strategy has been most extensively examined in the domain of ECG interpretation (Ark et al. 2006, 2007; Eva et al. 2007). A series of studies have demonstrated benefit to encouraging novice psychology students to use a dual-processing strategy when diagnosing ECGs over either strategy in isolation or lack of explicit guidance regarding how to reason through cases.

While these studies suggest that explicit instruction to use dual-processing may be a viable strategy to reduce error, it is unclear whether this benefit translates to more authentic and complex clinical contexts. Importantly, none of these studies examined clinicians with formal medical training. In addition, ECG interpretation is a visual task, where all relevant information is immediately available. However, most clinical decisions are more complex, requiring clinicians to simultaneously collect and interpret relevant information and to use those interpretations to guide subsequent data gathering. While dual-processing may improve clinicians’ decisions when all diagnostic information is available, its effect on a clinician’s ability to collect all relevant information is unknown.

One complex task requiring clinicians to actively collect information and decide on a diagnosis is cardiac physical examination. This task is readily adaptable to studying the pedagogical effects of a dual-processing instruction and is applicable to both novice and clinically experienced learners. The process of cardiac physical examination has features of value to both analytic reasoning (requiring identification and interpretation of specific findings) and non-analytic reasoning (through matching clinical presentations to previously encountered patients). The development of cardiac patient simulators allows the standardization of clinical findings in a manner that is suitable for research purposes.

Previous research using a cardiac simulator has shown that provision of a clinical history, potentially facilitating non-analytic pattern recognition, improved diagnostic accuracy among internal medicine residents (Sibbald et al. 2011). However, when the clinical history was misleading, residents were prone to diagnostic error, reflecting a potentially detrimental over-reliance on non-analytic pattern recognition (Sibbald and Cavalcanti 2011). Interestingly, the effects of the clinical history were restricted to the subset of trainees able to generate correct hypotheses from the clinical history. This may reflect the importance of nonanalytic processing in hypothesis formation.

The current study examines whether verbal prompting to use a dual-processing strategy when reasoning through clinical cases is of benefit to a spectrum of learners for cardiac physical examination. Because instruction can never guarantee adoption of a particular reasoning strategy and because we do not want to inflate the likelihood of observing an effect by limiting participants to strategies they might not normally adopt, we chose to compare dual-processing instructions to a control group that was encouraged to proceed with diagnosis in their usual fashion.

## Methods

Three separate but related experiments were conducted in three different populations along a gradient of expertise: experienced cardiology fellows (8–11 years of formal medical training), intermediate medical residents (6 years of formal medical training), and novice medical students (3 years of formal medical training). A similar experimental protocol was used with each cohort. Here we offer a generic overview of the design features consistent across all experiments. Specific modifications for each experiment will be outlined below (see Table 1).

**Table 1:** Methodological differences between the three experiments

Experiment number	1. Experienced trainees	2. Intermediate trainees	3. Novice trainees
Population	Twenty-six cardiology subspecialty residents	Thirteen internal medicine residents	Twenty-five third year medical students
Instruction phase diagnoses	Normal findings with physiologic third heart sound Aortic stenosis Acute mitral regurgitation Aortic coarctation Pulmonic stenosis Ventricular septal defect Acute aortic regurgitation Patent ductus arteriosus	Normal findings with a physiologic third heart sound Aortic stenosis Mitral regurgitation Hypertension with S4 Aortic sclerosis Ventricular septal defect Mitral stenosis Atrial septal defect	Normal findings with a physiologic third heart sound Aortic stenosis Mitral regurgitation Hypertension with S4 Aortic sclerosis Aortic regurgitation Mitral stenosis Aortic stenosis
Testing phase lesions	Aortic coarctation Ventricular septal defect Pulmonic stenosis Patent ductus arteriosus	Aortic stenosis Mitral regurgitation Hypertension with S4 Atrial septal defect	Aortic stenosis Mitral regurgitation Normal findings with a physiologic third heart sound Aortic regurgitation
Verbal bias in testing phase	For half of lesions	For all lesions	For half of lesions
Use of novel versions in testing	None	For 2 lesions (aortic stenosis and mitral regurgitation)	For 2 lesions (aortic stenosis and mitral regurgitation)
Timing of testing	Immediately following learning phase	Up to 48 h after learning phase	Up to 48 h after learning phase

### Intervention

Participants were randomized to receive one of two verbal instructions: dual-processing versus an undirected strategy. The dual-processing instruction was adapted from previous ECG based studies (Ark et al. 2006, 2007; Eva et al. 2007): “Cardiac presentations often sound similar to cases you have encountered before, such as the cases we reviewed during the last hour. Trust that sense of familiarity. However, be sure to avoid being trapped by your initial diagnostic hypotheses by carefully considering whether or not each of the specific individual findings is consistent with your diagnosis.” The undirected strategy prompt was: “Please conduct a cardiac physical exam as you would examine any patient you encounter in your clinical practice.”

### Design

Participants completed three phases: instruction, practice and testing. During the instruction phase, a facilitator introduced several cardiac physical diagnoses on a cardiopulmonary simulator, pointing out the key features of each diagnosis to a group of four or five participants over 1 h. Each participant was equipped with a wireless stethoscope and given the opportunity to identify all key features on the cardiopulmonary simulator. The simulator was a full-sized mannequin simulating realistic findings including pulses, jugular venous pressure and precordial impulses and murmurs. A 1-h practice phase immediately followed, during which the same disorders were presented in random order. Participants were asked to identify each diagnosis as a group and to practice their auscultation skills using the cognitive processing instruction to which they were assigned. During the testing phase, participants were individually tested on four diagnoses, again using the cognitive processing instruction to which they were assigned. In experiment 1, the test phase occurred immediately after practice whereas in experiments 2 and 3 it was scheduled on a different day due to time constraints.

During each of the three phases, the verbal prompt (either dual-processing or undirected) was read verbatim by the facilitator prior to demonstration, practice or testing of each lesion.

### Outcomes

For each of the four test cases, participants were asked to list diagnoses they thought to be probable and to assign a probability to each ranging from 1 to 100 % such that all diagnoses thought possible would sum to 100 %. If the correct diagnosis was not listed it was assigned a probability of 0 %. Subsequently, participants were asked to list key features used in reaching their diagnosis. In some cases a misleading history was also presented. We anticipated that dual-processing would lead to higher diagnostic accuracy, particularly if a misleading history accompanied the simulation. We also expected the likelihood of such an effect to be greater in less experienced trainees relative to their more experienced counterparts.

Ethical review was obtained from review boards at both participating institutions.

## Experiment 1: experienced cardiology fellows

### Methods

Twenty-six cardiology subspecialty residents voluntarily participated: 7 in postgraduate year (PGY) 4, 9 in PGY 5 and 10 in PGY 6. Eight diagnoses were presented in the instruction and practice phase in random order. Four were chosen to reflect lesions not commonly encountered in practice, and thus likely to offer meaningful learning to this group of experienced trainees: aortic coarctation, ventricular septal defect, pulmonic stenosis and patent ductus arteriosus. These were paired with four more commonly encountered lesions with similar findings: normal findings with physiologic third heart sound, aortic stenosis, acute aortic regurgitation, and acute mitral regurgitation.

The four less common diagnoses were used during the testing phase. A verbal bias meant to lead participants to the more common (but incorrect) diagnostic alternative was offered for two of the four diagnoses, chosen at random: e.g., “one of your colleagues thought this was aortic stenosis”. This was done to avoid ceiling effects that might arise with experienced trainees and because a dual-processing strategy has been shown to protect against diagnostic error arising from such biasing (Eva et al. 2007). After listing their differential diagnosis and assigning probability ratings for each test case, participants were asked to re-visit the cases and write down the key features that influenced their decision-making. Key features were classified and summed in four mutually exclusive groups: supportive of the correct diagnosis, supportive of the alternative diagnosis, supportive of neither diagnosis and supportive of both diagnoses.

### Results

Mean probabilities assigned to the correct diagnosis (with standard errors) are illustrated in Table 2. The correct diagnosis was assigned an average probability of 81.3 ± 4.2 % in the undirected instruction arm and 73.8 ± 4.7 % in the dual-processing instruction arm (*F*_1,103_ = 1.16, *p* = 0.25). There was no significant effect of biasing towards an inaccurate diagnosis (*F*_1,103_ = 1.05, *p* = 0.30) with the correct diagnosis assigned a probability of 80.6 ± 4.1 % in biased cases versus 73.9 ± 4.8 % in non-biased cases. There was no interaction between instruction and the provision of a verbal bias suggesting the alternate diagnosis (*F*_2,103_ = 0.09, *p* = 0.77). However, there was a significant interaction between the instruction provided and the diagnosis examined (*F*_3,103_ = 4.12, *p* = 0.01). Provision of dual-processing instructions was associated with lower probability of the correct diagnosis for aortic coarctation relative to undirected instructions (mean difference 38.1, *p* = 0.03). No differences were found for the other diagnoses (all *p* values >0.4). Finally, there was no interaction observed between the instruction provided and PGY (*F*_2,103_ = 0.32, *p* = 0.73; data not shown).

**Table 2:** Mean probabilities ± standard errors assigned to the correct diagnosis in experiment 1 (experienced cardiology fellows)

	Undirected instruction (*n* = 12)	Dual-processing instruction (*n* = 14)	Difference (Dual–undirected)
Overall mean	81.3 ± 4.2	73.8 ± 4.7	−7.5
Diagnosis tested (*n*)			
Aortic coarctation (26)	86.3 ± 8.1	48.2 ± 13.4	−38.1
Ventricular septal defect (26)	67.8 ± 10.1	77.9 ± 7.2	10.1
Pulmonic stenosis (26)	84.8 ± 6.8	77.0 ± 7.2	−7.8
Patent ductus arteriosus (26)	86.2 ± 8.3	92.3 ± 2.4	6.1
Bias (*n*)			
None (52)	84.2 ± 5.6	77.5 ± 5.9	−6.7
Alternative diagnosis (52)	78.3 ± 6.3	70.2 ± 7.3	−8.1

Feature identification did not differ between the undirected and dual-processing instructions whether features were supportive of the correct diagnosis (2.5 vs. 2.6), supportive of the alternative diagnosis (0.5 vs. 0.7), supportive of either diagnosis (1.5 vs. 1.7), or supportive of neither diagnosis (2.6 vs. 2.4), all *p* values >0.4. Similarly, feature identification did not differ for diagnoses presented with or without bias (all *p* values >0.2).

## Experiment 2: intermediate trainees (medical residents)

### Methods

Thirteen internal medicine residents from a single institution voluntarily participated. Given the small sample size, this experiment biased all participants towards the alternate diagnosis during the test phase for each test case to enhance the likelihood of detecting an effect of the instructional condition. The test phase was scheduled within 48 h of the instruction/practice phases rather than immediately after instruction due to time constraints.

For this group of learners, four more common and/or clinically important cardiac diagnoses were chosen as test cases: aortic stenosis, hypertension with S4, atrial septal defect and mitral regurgitation. During the instruction/practice phases four additional diagnoses were also presented: aortic sclerosis, normal findings with a physiologic third heart sound, ventricular septal defect and mitral stenosis. Features were scored as supportive of the correct diagnosis, supportive of either the correct or alternate diagnosis and supportive of neither diagnosis.

In order to assess whether dual-processing instructions would aid identification in another version of the same lesion, a novel version of aortic stenosis and mitral regurgitation were programmed into the cardiopulmonary simulator. While both versions contained the key features of the diagnosis, they varied on minor attributes not relevant to the diagnosis. As a result, the test phase in this study consisted of two cases that were literal replications of cases seen during the learning/practice phase of the study and two that were unfamiliar versions of the diagnosis used.

### Results

Mean probabilities assigned to the correct diagnosis (with standard errors) are illustrated in Table 3. The correct diagnosis was assigned an average probability of 70.3 ± 7.1 % in the undirected instruction arm and 82.4 ± 8.6 % in the dual-processing instruction arm (*F*_1,33_ = 1.1, *p* = 0.3). The interaction between the instruction provided and the diagnosis given was non-significant (*F*_3,33_ = 0.7, *p* = 0.6) although dual-processing instructions were associated with higher probabilities for three of the four diagnoses with mean differences greater than those typically found in this literature.

**Table 3:** Mean probabilities ± standard errors assigned to the correct diagnosis in experiment 2 (medical residents)

Diagnosis tested (*n*)	Case novelty	Undirected instruction (*n* = 6)	Dual-processing instruction (*n* = 7)	Difference (Dual–undirected)
Overall mean		70.1 ± 7.1	82.4 ± 8.6	12.3
Aortic stenosis (13)	New version	86.4 ± 14.1	72.8 ± 17.2	−13.6
Mitral regurgitation (13)	New version	62.7 ± 14.1	88.4 ± 19.9	25.7
Hypertension (13)	Same version	63.2 ± 13.0	78.7 ± 14.1	15.5
Atrial septal defect (13)	Same version	68.7 ± 14.1	89.8 ± 17.2	21.1

The number of features identified was not different in the dual-processing group relative to the undirected group whether features were supportive of the diagnosis (5.0 vs. 4.3), supportive of either (3.1 vs. 2.5) or supportive of neither (1.2 vs. 1.1), *p* for all >0.1.

## Experiment 3: novice trainees (medical students)

### Methods

Twenty-five 3rd year medical students from a single institution were recruited over the course of one academic year. Given that the previously published benefits of dual processing instruction were seen in novices, this group was expected to be the most likely to benefit from dual-processing instruction.

The four diagnoses assessed in the test phase were aortic stenosis, normal findings with a physiologic third heart sound, aortic regurgitation and mitral regurgitation. These were paired during the instruction and practice phases with aortic sclerosis, left ventricular hypertrophy with audible fourth heart sound, mitral stenosis and aortic stenosis.

In the test phase, participants were randomly biased to the paired alternate diagnosis in two out of the four cases as in Experiment 1. Features were recorded as supportive of the diagnosis, supportive of either the correct or alternate diagnosis and supportive of neither. Identical to Experiment 2, a novel version of aortic stenosis and mitral regurgitation were programmed into the cardiopulmonary simulator during the test phase. Also identical to Experiment 2 is the presence of up to a 48-h delay between instruction/practice and test to accommodate scheduling requirements.

### Results

Mean probabilities assigned to the correct diagnosis (with standard errors) are illustrated in Table 4. The correct diagnosis was assigned an average probability of 67.5 ± 5.6 % in the undirected instruction arm and 68.5 ± 5.7 % in the dual-processing instruction arm. The main effect was not statistically significant (*F*_1,78_ = 0.02, *p* = 0.9). There was no significant interaction between instruction and case (*F*_1,78_ = 0.8, *p* = 0.51).

**Table 4:** Mean probabilities ± standard errors assigned to the correct diagnosis in experiment 3 (medical students)

		Undirected instruction (*n* = 12)	Dual-processing instruction (*n* = 13)	Difference (Dual–undirected)
Overall mean		67.5 ± 5.6	68.5 ± 5.7	1.0
Diagnosis tested (*n*)	Case novelty			
Aortic stenosis (24)	Same version	52.5 ± 11.1	65.1 ± 11.1	12.6
Normal with S3 (22)	Same version	60.9 ± 11.5	71.5 ± 11.5	10.6
Aortic insufficiency (24)	New version	71.9 ± 11.1	71.1 ± 11.1	−0.8
Mitral regurgitation (24)	New version	84.8 ± 10.8	66.3 ± 11.9	−18.5
Bias (*n*)				
None (49)		78.9 ± 7.8	74.0 ± 7.9	−4.9
Alternative diagnosis (45)		56.1 ± 8.0	63.0 ± 8.3	6.9

The diagnostic probability was lower for those diagnoses presented with a bias (59.6 ± 5.8 %) versus those presented without a biasing diagnosis (76.4 ± 5.5 %, *F*_1,78_ = 4.5, *p* = 0.04). While the effect of bias appears more pronounced among participants given undirected reasoning instructions (56.1 ± 8.0 % with bias vs. 78.9 ± 7.8 % without, difference = 22.8, *F*_1,40_ = 5.4, *p* < 0.03) compared to those with dual-processing instructions (63.0 ± 8.3 % with bias vs. 74.0 ± 7.9 % without, difference = 11.0, *F*_1,38_ = 0.7, *p* = 0.39), the overall interaction was non-significant (*F*_1,78_ = 0.6, *p* = 0.45).

There were no differences in the features identified between the undirected or dual-processing group whether features were supportive of the correct diagnosis (1.9 vs. 1.8), supportive of either diagnosis (3.2 vs. 3.1) or supportive of neither diagnosis (2.3 vs. 1.7; *p* > 0.2 in all cases). There were no differences in the features identified between diagnoses presented with bias and those presented without bias (*p* for all >0.3).

## Discussion

To our knowledge, this is the first study to examine the impact of dual-processing instruction on the learning process of medical trainees. Despite three different experiments in three groups of trainees, verbal instructions to apply dual-processing did not result in improved overall diagnostic accuracy. While no signal of benefit was present among advanced learners, we cannot rule out a significant interaction between content and dual-processing instructions or between instruction and bias among intermediate and novice trainees.

Several aspects of this intervention that may have led to our findings need be addressed: application to a complex skill, content dependence, dependence on expertise level, and the strength of the instruction.

### Application to a complex skill

Prior successes of reasoning instruction interventions were all within the realm of visual diagnosis (Ark et al. 2006, 2007; Hatala et al. 1999; Regehr et al. 1994), where the cognitive load is constrained to a visual space. Cardiac physical exam, similar to many clinical problems, involves the integration of multimodal perception. This added integration likely results in an inherently higher intrinsic and extrinsic cognitive load on the clinician (van Merrienboer and Sweller 2010).

The intrinsic cognitive load in cardiac diagnosis may be too great for clinicians to also allot working memory to deliberately guiding their reasoning practices. Clinicians may deal with the cognitive load by automating the more mundane components of the task. Further, use of procedural scripts may result in less opportunity to modulate the cognitive processes as directed in the reasoning instructions.

### Content dependence

Instructions to promote dual-processing had a varied effect on diagnostic accuracy based on the content of the station. Diagnosis of the rarest lesion in each set was harmed by instructions for dual-processing (aortic coarctation in the experienced learners and atrial septal defect in the intermediates). In contrast, there was a suggestion of benefit in diagnosing common lesions among intermediates and overcoming bias among novices.

The nature of the content studied should be carefully considered in future research into dual-processing instructions. Consistent with this idea, Chamberland et al. (2011) have recently reported that case familiarity appears to have an influence on the benefit observed from having students engage in self-explanations relevant to their diagnostic performance. Sampling of cases needs to include a mix of rare and common, and likely a larger sample of total cases, in order to better understand the interaction of individual diagnoses and reasoning strategy and to reduce the amount of within subject variance that added noise to these experiments. The previously published ECG studies routinely used 14–20 test cases to overcome content dependence.

Importantly, the simulator has a finite set of abnormalities. While the findings themselves are authentic, the known limited repertoire of the simulator generates a problem space that is much narrower than clinicians face in routine practice. As a result, we may underestimate the need for dual-processing in clinical practice by using a research oriented model that allows non-analytic pattern recognition around a few key features with little variability in presentation.

### Experience level

Prior success of dual-processing instruction was found among absolute novices (Ark et al. 2006, 2007; Eva et al. 2007), but not among intermediate level trainees (Sibbald and de Bruin 2011). No overall effect of dual-processing instruction was found in our study among formal medical trainees across a range of expertise levels. However, as in the studies reported with absolute novices (Eva et al. 2007), dual processing instructions may have helped out relatively novice medical students overcome the biasing influence of an inaccurate diagnostic suggestion.

Diagnoses were specifically chosen to challenge each level of trainee so that the effect of dual-processing instructions on learning could be assessed. Our assumption was that the directive strength of verbal prompting would be greatest when faced with new material. However, the high probabilities assigned to the correct diagnosis across all levels suggested that most were familiar with these diagnoses.

Of note, even the novice medical trainees included in this study would have spent up to 100 h in the first several years of the medical school conducting cardiac physical exams. As a result, their exposure to the process is substantial. Given this experience, many may have already been taught to use a dual-processing approach or intuitively balanced their reasoning strategies thus negating any intervention effect. This is suggested by the similarity in the number of features identified by both reasoning groups, suggesting a similar cognitive strategy used by both. Alternatively, interfering with the reasoning process at a more advanced stage of clinical development may be detrimental as suggested by the expertise literature, which shows that manipulating the thought process of experienced clinicians may lead to an expertise reversal effect or paradoxical worsening of performance (Kalyuga 2007). Given the rapidity with which medical knowledge and skill is acquired in formal medical education and the speed with which previously seen cases have been found to begin influencing judgments on novel cases, finding absolute novices in a medical training setting may be difficult. Further study might target more novice first year medical students.

### Directive strength of the intervention

Because cardiac physical examination is used on a daily basis, all of our participants may have an entrenched routine. As a result, it may be difficult to modify with a single verbal instruction. We incorporated both a learning and practice phase to encourage the use of the reasoning instruction; a successful approach in previous studies (Ark et al. 2006, 2007; Eva et al. 2007). However, we did not measure reasoning strategy directly, and cannot be sure whether this approach was effective in our context. Additionally, the test phase was delayed in these experiments for the groups most expected to reveal an influence of reasoning instruction (i.e., the more novice trainees). This was done for the sake of making data collection feasible. However, we may have missed an immediate benefit of dual-processing instruction among novices and intermediates. Nevertheless, it suggests, at a minimum, that if there was an effect of instruction it was short lived. Further work is needed to explore how to strengthen cognitive interventions to more effectively modulate behavior of more experienced clinicians.

### Limitations

In addition to the methodological issues outlined above, several other limitations should be mentioned. First and foremost, the number of trainees was small. Estimated power to detect a 20 % difference in probability with dual-processing was 0.88, 0.60 and 0.87 among experienced, intermediate and novice trainees (Lenth 2007). The study was underpowered to detect smaller effects, which may be educationally important. In addition, only four lesions could be tested because of the time constraints involved, which differs from the twenty ECGs used in the previous positive studies (Ark et al. 2006, 2007; Eva et al. 2007). Second, several trainees struggled in assigning diagnostic probability ratings; some provided probabilities that summed to >100 %. While this is a common approach to assessing decision making, it is not without important limitations. Trainees may disproportionately weight small or large percentages and may have different concepts of what percentage constitutes sufficient diagnostic certainty for ascribing a diagnosis to a patient or providing a therapy. Third, exaggerated findings on the simulator may have decreased the need and efficacy of dual-processing instruction. Finally, the group process implemented in the instructional and practice phases of this research to overcome the feasibility issues inherent in providing individualized training (as was done in the ECG studies) may have lessened the strength of the intervention. If some individuals in the group mentioned similarity to past examples and others deliberately focused on the feature presentations, then even those in the undirected group would have experienced dual processing practice.

### Implications

The application of dual-processing strategies to reduce cognitive error requires further investigation. Its blind application to clinical medicine needs to be tempered by these results. As applied to cardiac auscultation on a cardiopulmonary simulator, instructions to encourage dual-processing were not successful in reducing cognitive error.

Future study should focus on the utility of promoting dual-processing among novices while also considering content dependence, intervention strength, the complexity of the skill required for diagnosis, and the role that group practice might play in overcoming the biases of individuals.
